# Association between Work Environment and Outcomes of Primary Care Nurse Practitioners Caring for Dementia Patients

**DOI:** 10.1093/geroni/igaf122.2419

**Published:** 2025-12-31

**Authors:** Lusine Poghosyan, Maura Dougherty, Kyle Featherston, Jianfang Liu, Josh Porat-Dahlerbruch

**Affiliations:** Columbia University, New York, New York, United States; Columbia University, New York, New York, United States; Columbia University School of Nursing, New York, New York, United States; Columbia University Irving Medical Center, New York City, New York, United States; University of Pittsburgh School of Nursing, Pittsburgh, Pennsylvania, United States

## Abstract

Nurse practitioners (NPs) play a key role in caring for patients with dementia in primary care practices and can expand the dementia workforce capacity. Yet, NPs often practice in poor work environments, lacking support and resources. We examined NP work environments and job outcomes (i.e., burnout, job dissatisfaction, and intent to leave the job in the coming year) among primary care NPs providing dementia care. We surveyed a national U.S. sample of NPs from primary care practices delivering dementia care in 2021. In total, 968 NPs from 847 practices responded. The work environment was measured using the Nurse Practitioner Primary Care Organizational Climate Questionnaire and its four subscales. While 92% of NPs reported being satisfied with their job, 35.8% reported experiencing burnout, and 21.3% reported intent to leave their current job. A one-unit increase in the NP-Administration Relations subscale score was associated with 64% decreased odds of burnout (cumulative OR = 0.36; p = < 0.0001), a three-fold increase in the odds of job satisfaction (cumulative OR = 3.42; p = < 0.0001), and 75% lower odds of intent to leave (OR = 0.25; p = < 0.0001). A one-unit increase in the Independent Practice and Support score was associated with 42% lower odds of burnout (cumulative OR = 0.58; p = 0.04) and 71% increase in the odds of having higher job satisfaction (cumulative OR = 1.71; p = 0.04). Our findings suggest that working relationships with administrators and practice support are key for promoting NP outcomes, potentially improving dementia care quality.

